# Taste-Masked Microparticles of Sodium Warfarin Prepared by Hot Melt Extrusion and Thermal Postprocessing

**DOI:** 10.3390/pharmaceutics18050582

**Published:** 2026-05-08

**Authors:** Paula Kaufelde, Andrey Aniskevich, Jevgenijs Sevcenko, Marta Žogota, Zoltán Márk Horváth, Valentyn Mohylyuk

**Affiliations:** 1 Leading Research Group, Faculty of Pharmacy, Riga Stradiņš University, LV-1007 Riga, Latvia; paula.kaufelde@rsu.lv (P.K.);; 2 Institute for Mechanics of Materials, University of Latvia, LV-1004 Riga, Latvia

**Keywords:** Kollicoat^®^ Smartseal, Eudragit^®^ E PO, taste masking, hot melt extrusion, pellets, personalization

## Abstract

**Background/Objectives.** Warfarin possesses a bitter taste and requires a personalized dose in the range of 4.5 to 77 mg per week. This study investigated the potential for personalizing warfarin dosing by developing taste-masked matrix pellets. Pellets, supposedly, can be counted and orally administered in the required quantity to obtain the required dose. **Methods.** The study evaluated the effects of drug load (10, 20, and 30 wt.%) on the duration of thermal postprocessing (to achieve the desired aspect ratio) and drug release. The warfarin sodium clathrate was characterized by determining its pKa value and dissolution kinetics in water, stomach-simulated media (0.1 M HCl, pH 1.2), and mouth-simulated media (phosphate-buffered solution (PBS), pH 6.8). A solid dispersion of warfarin sodium clathrate with Kollicoat^®^ Smartseal 100 P or Eudragit^®^ E PO was prepared using hot melt extrusion (HME). The mechanical properties of extruded filaments were characterized by measuring their elastic modulus. The microparticles (1–4 mm in length) prepared with filament cut pelletizing were thermally treated to produce ‘smoothened’ particles, which were analyzed with optical microscopy and drug release testing. **Conclusions.** Microparticles with smoothened edges and an aspect ratio close to one are expected to improve mouthfeel and potentially patient compliance. No drug release was observed in mouth-simulated media, which indicated applicability to the taste masking of the microparticles. The proposed thermal treatment of HME microparticles, exemplified in this study, is a novel concept with underexplored potential in preparing taste-masked matrix pellets and dose personalization.

## 1. Introduction

Warfarin, a coumarin derivative and indirect-acting anticoagulant, is a vitamin K antagonist. The use of warfarin is primarily for the prophylaxis and treatment of deep venous thrombosis (DVT) and is commonly prescribed to manage conditions associated with recurrent thromboembolic events and related complications [1,2,3].

**Figure 1:**
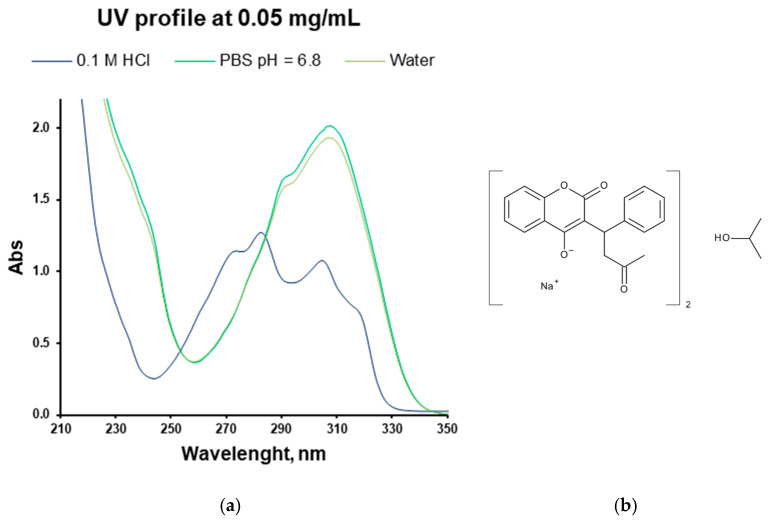
UV profiles of 0.05 mg/mL warfarin sodium clathrate solution in 0.1 M HCl, water and PBS pH = 6.8 (**a**); structure of warfarin sodium clathrate (**b**) [4].

Warfarin is classified as a class II drug in the BCS system [4], with the experimentally estimated pKa in the range from 4.85 to 5.15 [5]. Due to warfarin’s poor solubility in aqueous solutions, commercially available dosage forms use the crystalline sodium clathrate form, consisting of warfarin sodium salt and isopropyl alcohol in a 2:1 molar ratio [6] (Figure 1), possessing a monoclinic space lattice [7].

The primary challenges and limitations in warfarin therapy are associated with hy-percoagulable conditions [8] or increased bleeding risk due to improper dosing related to genetic polymorphisms and warfarin’s very narrow therapeutic window [2,5]. Patients with these genetic variants typically require lower doses, associated with an increased risk of bleeding if standard doses are used [2]. The dose for each patient for anticoagulation varies in the range between 4.5 and 77 mg per week. To achieve the desired single dose, patients may need to combine multiple tablets or split tablets, which reduces compliance and increases the risk of side effects [9]. Frequent dose adjustments are often required, leading to the need for dose personalization [2].

Warfarin is available in specific, limited, commercially available dosage forms. Pri-marily, warfarin is commercially available in the form of oral tablets, with dosage ranging from 1 mg to 10 mg (Coumadin^®^) [10]. However, it is also available as an oral suspension and can be administered intravenously [2], as well as in lyophilized powder for injection. Tablets are the most common dosage form; however, liquid formulations are available for patients who have difficulty swallowing, such as children or the elderly, as oral admin-istration in such cases can lower compliance [10,11].

Splitting tablets or taking multiple tablets is a method of personalized dosing. How-ever, this approach may lack precision [9]. As an alternative approach, multi-unit systems such as mini-tablets or pellets can be considered [12]. Depending on the required dose, the number of mini-tablets or pellets can be adjusted accordingly [13].

Another limitation of oral formulations is warfarin’s bitter taste [14,15]. Attempts to prepare oral liquid taste-masking formulations were previously undertaken [14]. However, one of the most effective ways to prevent a bitter taste is to prevent bitter drug dissolution in the oral cavity. Various polymers with pH-dependent solubility can be used for this purpose, like acrylate-based copolymers [16]. Examples involve aminoalkyl methacrylate copolymer (Eudragit^®^ E) [17] or methyl methacrylate and diethylaminoethyl methacrylate copolymer (Kollicoat^®^ Smartseal 100 P) [12,18]. Another option is polyvinylacetal diethylaminoacetate (AEA) [19]. Effectively reducing bitterness, these polymers exhibit low solubility at the pH of the oral cavity (pH 5.8–7.4), but they are soluble at the pH of the stomach (pH 1–3.5) [16].

For taste masking, dosage forms can be implemented in the coated form [4] or matrix system using a polymer with pH-dependent solubility [16]. In the case of a matrix system, solid dispersion can be obtained by hot melt extrusion (HME), and taste-masked microparticles can be obtained by HME filament cut pelletizing [20].

Taste masking of bitter drugs using functional polymers and HME is currently under development [21]. For example, the effect of taste-masking polymer type (Kollicoat^®^ Smartseal 100 P, Eudragit^®^ EPO, and Kollicoat^®^ MAE 100-55) on the properties of theophylline-containing (10–30 wt.%) pellets was investigated in the context of face-cut pelletized matrix pellets for pediatric application [22]. But cutting the filament typically results in microparticles with sharp edges, which poses discomfort and risk of oral soft tissue damage [23]. There are a significant number of scientific references on using taste-masking polymers (such as Eudragit^®^ E PO or Kollicoat^®^ Smartseal 100 P) for the preparation of taste-masked matrix pellets by HME and face-cut pelletization. Nevertheless, to the best of our knowledge, there is only one literature source reporting smoothening Eudragit^®^ E PO-based particle sharp edges by thermal postprocessing [24]. Microscopic images of pelletized filament particles before and after treatment (at 85–95 °C) in the aqueous media showed the ability of the thermal treatment to change the aspect ratio and to smooth the cut side surfaces. The effect of temperature and the aqueous heat treatment duration on initial pelletized filament particles showed an increase in their aspect ratio difference (ΔAR) with an increase in temperature and treatment duration. The treatment-induced ΔAR was more pronounced for particles with an initial diameter of 1.01 ± 0.06 mm and initial length of 2.34 ± 0.20 mm than for particles with an initial diameter of 1.51 ± 0.09 mm and initial length of 2.93 ± 0.18 mm (Av. ± SD; n = 10). In addition, the thermal treatment with hot air showed the same tendency of ΔAR change as the aqueous thermal treatment [24]. It is known that applying thermal treatment near the polymer’s glass transition temperature enables elastic deformation of the polymer macromolecules [25]. By experimentally optimizing the duration of thermal treatment, microparticles with an aspect ratio close to one can be produced, resulting in shapes that more closely resemble spheroids or mini-tablets [24,26,27]. Thus, the applicability of other polymers for this purpose was not investigated.

This study aimed to investigate the potential for warfarin dose personalization through the preparation of taste-masking Kollicoat^®^ Smartseal 100 P and Eudragit^®^ E PO matrix pellets via hot melt extrusion and thermal postprocessing. During the study, the effects of drug load (10, 20, and 30 wt.%) on the duration of postprocessing thermal treatment (to achieve the desired aspect ratio) and on drug release were examined.

## 2. Materials and Methods

### 2.1. Materials and Reagents

The following were obtained: warfarin sodium clathrate (batch # WA22003; Alchymars Icm SM Private Ltd., Tamil Nadu, India) with a warfarin and isopropyl alcohol molar ratio of 2:1 [4]; methyl methacrylate (MMA) and diethylaminoethyl methacrylate (DEAEMA) copolymer (Kollicoat^®^ Smartseal 100 P; BASF SE, Ludwigshafen, Germany); dimethylaminoethyl methacrylate (DEAEMA), butyl methacrylate (BMA), and methyl methacrylate (MMA) copolymer (Eudragit^®^ E PO; Evonik Operations GmbH, Darmstadt, Germany); microcrystalline cellulose spheroids (Celphere™ CP-507 (500–710 μm); Asahi Kasei Co., Tokyo, Japan); glass beads (60 mesh/250 μm; BDH Laboratory Supplies, Poole, UK); and hydrochloric acid (HCl), sodium hydroxide pellets (NaOH), phosphoric acid (H_3_PO_4_), and potassium phosphate monobasic (KH_2_PO_4_; Merck KGaA, Darmstadt, Germany).

### 2.2. Warfarin Sodium Clathrate Solubility Determination and UV Quantification

The excess of warfarin sodium clathrate was weighed in 100 mL volumetric flasks. Solubility was determined using a shake flask method under ambient conditions with a magnetic stirrer at 200 rpm for two days. Solutions were filtered (Filtropur S 0.45; Sarstedt AG & Co. KG; Nümbrecht, Germany), and equilibrium solubility was determined spectrophotometrically (Shimadzu UV-1900i; Shimadzu, Kyoto, Japan) at a wavelength of 260 nm in PBS (C = (Abs. − 0.0204)/7.0751; R^2^ = 0.9999), 340 nm in water (C = (Abs. − 0.0072)/1.5819; R^2^ = 0.9991) and 307 nm in 0.1 M HCl solution (C = (Abs. − 0.0025)/30.148; R^2^ = 0.9999).

### 2.3. Observation of the UV Absorbance and pH Change During the Dissolution of the Raw Drug Substance

Dissolution kinetics data were obtained by dissolving an excess of warfarin sodium clathrate in cuvettes containing 0.1 M HCl, water, and PBS (pH 6.8). The samples were stirred with a magnetic stirrer at 200 rpm. The absorbance spectrum of each sample was measured every 5 min for 90 min using a UV spectrophotometer (Shimadzu UV-1900i; Kyoto, Japan) to obtain UV profiles. Absorbance at 450 nm was subtracted from the spectrum data to eliminate the influence of turbidity. A wavelength of 283 nm was chosen for analyzing dissolution kinetics in 0.1 M HCl solutions, while 372 nm was used in water and PBSs.

A corresponding pH change in warfarin sodium clathrate-containing solutions was determined by weighing and dissolving an excess of the warfarin sodium clathrate in a glass vial in each medium. Solutions were stirred with a magnetic stirrer at 200 rpm, and pH was measured using a pH/conductivity meter (SD230KIT; Mettler Toledo AG, Columbus, OH, USA) for 90 min uninterruptedly.

### 2.4. Potentiometric Titration

The pKa was determined using a potentiometric titrator (AT-710S, Kyoto Electronics Manufacturing Co., Ltd., Kyoto, Japan) through alkaline titration. A total of 7.3 mg of warfarin sodium clathrate was dissolved in a 100 mL volumetric flask. The sample was diluted 50 times, and three drops of 0.05 M HCl were added to lower the pH to approximately 4.1. The sample was then titrated with pre-standardized 0.025 M NaOH at 22.1 °C. The inflection (equivalence) point was determined by calculating the first derivative, and the pKa value was obtained using the pH at the half-equivalence point (based on the Henderson–Hasselbalch equation) [28].

### 2.5. Loss on Drying

Warfarin sodium clathrate was tested with thermogravimetric analysis (TGA; loss on drying). The accurately weighed sample of approx. 500 mg was exposed to a constant temperature of 180 °C or 190 °C and the weight loss was recorded (HX204; Mettler Toledo AG, Greifensee, Switzerland). Measurements were made in triplicate, and the results were presented as averages, including the standard deviations (n = 3; Av. ± S.D.).

### 2.6. Hot Melt Extrusion (HME)

A physical mixture containing 0, 10, 20, and 30 wt.% warfarin sodium clathrate (*w*/*w*) with Kollicoat^®^ Smartseal 100 P, as well as 20 wt.% warfarin sodium clathrate (*w*/*w*) with Eudragit^®^ E PO, was mixed using a DVD Developer ‘Interchangeable’ mixer (Comasa, Barcelona, Spain). Sieving was conducted with a sieve of 1 mm mesh size (AS 200 digit cA; Retsch GmbH, Haan, Germany). Twin-screw hot melt extrusion was carried out using a co-rotating twin-screw extruder (Thermo Fisher Pharma 11, Thermo Scientific™, Karlsruhe, Germany). The extruder was fed through a Pharma 11 Volumetric Twin-Screw Feeder (Thermo Scientific™, Karlsruhe, Germany). To obtain Kollicoat^®^ Smartseal 100 P filaments with 0, 10, 20, and 30 wt.% warfarin sodium clathrate and Eudragit^®^ E PO with 20 wt.% warfarin sodium clathrate, the mixtures were fed at 4.63 g/min and 2.69 g/min rates, respectively. The screws were equipped with conveying elements only (to avoid drug particle size reduction), and the rotation speed was set to 150 rpm. The horizontal split die with a 2 mm diameter was used. The glass transition temperatures of Kollicoat^®^ Smartseal 100 P and Eudragit^®^ E PO are 63 and 52 °C, while their degradation temperatures are above 220 °C [18,29]. Processing temperatures for Kollicoat^®^ Smartseal 100 P mixtures were maintained at 190 °C in seven zones, except for the feeding zone at 30 °C and the next zone at 70 °C. For Eudragit^®^ E PO mixtures, the temperature was maintained at 150 °C across all seven zones, except for the feeding zone, which was set to 30 °C, and the subsequent zone, set to 60 °C. The torque did not exceed 1.8 N·m. The first 3 g of extrudates from each batch were discarded. The filament was manually pulled from the extrudate, achieving a preferred diameter in the range of 0.3 to 1 mm. The filaments were kept in sealed plastic bags at room temperature [24,30].

### 2.7. Powder X-Ray Diffraction (pXRD)

The pXRD patterns were obtained by a MiniFlex600-C (Rigaku, Tokyo, Japan) diffractometer with a θ/2θ scan axis and 1D scan mode. The pXRD measurements were carried out at 40 kV and 15 mA in a step mode with a speed of 5°/min and a step size of 0.02° for 2θ = 3–60° with a total acquisition time of ~12 min for each sample. A silicon background was used with a spin of 10 rpm. Measurements were carried out in triplicate for each sample, and the averaged data were presented.

### 2.8. Attenuated Total Reflectance–Fourier Transform Infrared Spectroscopy (ATR-FTIR)

An FTIR-ATR study of the samples was performed on an FTIR spectrometer (Nicolete IS20, Thermo Scientific, Karlsruhe, Germany) using a diamond prism by scanning from 4000 to 400 cm^−1^, with a 2.0 cm^−1^ resolution and 100 scans per spectrum (the background was taken before each sample). Every graphically represented FTIR profile was obtained by averaging 3 spectra.

### 2.9. Elastic Modulus Measurement

Quasistatic: Tensile tests of the Kollicoat^®^ Smartseal 100 P filament samples were performed using a universal electromechanical testing machine, Zwick Roell Z 2.5 (Zwick & Roell, Ulm, Germany). The filament samples of 150 mm length were placed between metal clamps and carefully gripped. The diameter of each sample was measured using an electronic caliper with an accuracy of ±0.01 mm and was in the range of 0.3 to 1 mm. The grip-to-grip separation at the starting position was 100 mm, with a pre-load of 0.3 N and a test speed of 0.5 mm/min. Testing conditions were set at 23 °C and a relative humidity of 26%. Tensile strength and elastic modulus were calculated automatically using testXpert III software. The elastic modulus was calculated as the slope of the stress–strain curves in the 0.05–0.25% strain range (Equation (1)):

(1) $$E=\frac{\sigma }{\epsilon \;}$$

where ***E*** is the filament elastic modulus (GPa), ***σ*** is the stress (GPa), and ***ϵ*** is the strain.

The final results represent the mean of at least five measurements. All stress–strain curves were nearly linear with brittle failure and, for this reason, were not presented in the manuscript.

### 2.10. Thermal Treatment

Filaments (diameter 0.8 mm) with different drug loadings were cut into smaller particles with lengths of 4, 3, 2, and 1 mm. The particles were placed in a 1-L round-bottom flask containing 10 g of glass beads (60 mesh/250 μm, density of 2.4–2.8 g/cm^3^ [31,32]; BDH Laboratory Supplies, Poole, UK). Each particle length (n ≥ 11) was thermally treated near the polymer’s glass transition temperature (Kollicoat^®^ Smartseal 100 P Tg is approximately 65 °C [18], but Eudragit ^®^ E PO Tg is approximately 48–52 °C [29,33]) using a rotary evaporator (Chemtron Strike 380; Wiggens GmbH, Wuppertal, Germany) for 480 min or longer. The bath temperature was set at 80 °C, with a rotation speed of the flask at 20 rpm. The length and diameter of the particles before and after treatment were measured using an electronic caliper.

### 2.11. Optical Microscopy

The filaments were analyzed using an optical microscope (BA410E; Motic, Xiamen, China) equipped with a 50 W halogen lamp and Motic EC-H Plan objective lenses (4/0.1, 10/0.25, and 40/0.65). Images were captured using a MoticamProS5 Lite camera controlled by Motic Images Plus 3.0 software.

### 2.12. Particle Size Distribution: Laser Diffraction

Celphere™ CP-507 and warfarin sodium clathrate were vigorously manually mixed in an approximate weight proportion of 50:50 in a closed glass vial. The particle size distribution, as well as the D_10%_, D_50%_, and D_90%_, was determined by a laser diffraction particle size analyzer using an Aero S module for dry dispersions (Mastersizer 3000, Malvern Instruments, Malvern, UK) at the specified settings: a feed rate of 30–80%; a hopper gap of 1.0–1.5 mm; and an air pressure of 2.0 bar. Approximately 10–15 g of the sample was used for each repetition (n = 3) [4].

### 2.13. Drug Release from Microparticles

Thermally treated particles were tested in 500 mL of a phosphate-buffered solution (PBS, pH 6.8) or a 0.1 M HCl solution (pH 1.2), prepared according to the European Pharmacopoeia. The tests were conducted at 37 ± 0.5 °C using a USP II paddle apparatus (ATS Xtend™, SOTAX AG; Allschwil, Switzerland) operating at 100 rpm. A specific number of particles was weighed to achieve a warfarin dose of 2 mg/L. The particle’s surface area (SA) and volume (V) were calculated using cylindrical geometry, and the surface-area-to-volume ratio (SA/V) was determined by dividing SA by V (Equations (2) and (3)).

(2) $$SA=2\pi r\left(r+h\right),$$

Here, ***SA*** is the cylinder surface area (mm^2^), ***r*** is the radius (mm), and ***h*** is the height (mm).

(3) $$V=\pi \;r^{2}h,$$

Here, ***V*** is the volume of the cylinder (mm^3^), ***r*** is the radius (mm), and ***h*** is the height (mm).

The concentration of dissolved warfarin sodium clathrate was measured spectrophotometrically (Shimadzu UV-1900i; Kyoto, Japan) at a wavelength of 307 nm using the equation C = (Abs. − 0.0025)/30.148 with an R^2^ value of 0.9999 for 0.1 N HCl and C = (Abs. − 0.0006)/80.285 with an R^2^ value of 0.9994 for PBS derived from the calibration curves.

### 2.14. Statistical Assessment

A one-way ANOVA (analysis of variance) test was used to compare the means of two groups using the built-in possibilities of the current version of Excel (Microsoft 365; Redmond, Washington, DC, USA).

## 3. Results and Discussion

The UV profiles of warfarin sodium in water and PBS (pH 6.8) were similar. Warfarin exhibited strong absorbance across the 190–350 nm range. The UV absorption spectra of aqueous warfarin sodium clathrate solutions showed distinct peaks at 283 nm and 307 nm in 0.1 HCl, and at 315 nm in water and PBS, corresponding to π → π* transitions of the carbonyl group associated with keto-enol tautomerism [34] (Figure 1a).

**Figure 2:**
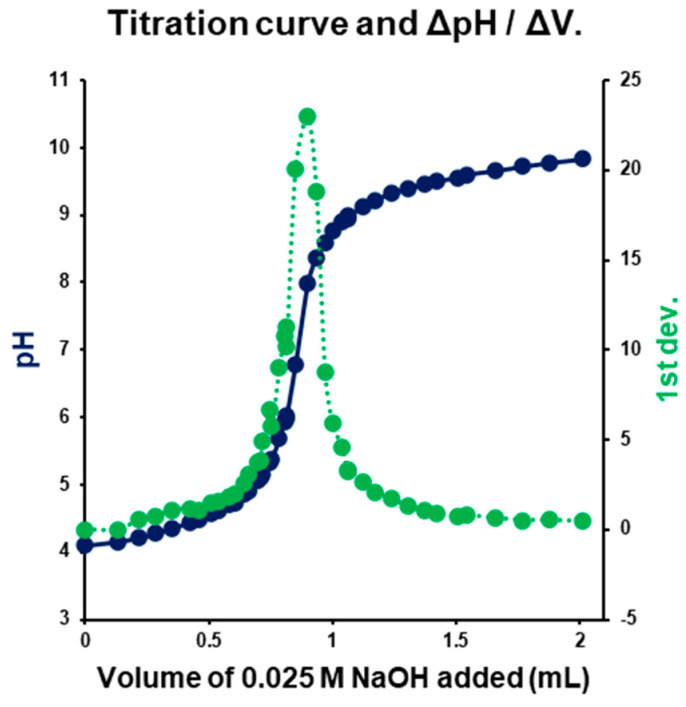
Alkaline titration curve and 1st derivative (ΔpH/ΔV).

The experimentally determined that the pKa value was 4.46 ± 0.05 (Av ± SD; n = 5) (Figure 2), which is lower than literature values (with pKa values between 4.85 and 5.15) [5]. It should be noted that the reported literature values were obtained using different experimental approaches, including spectrophotometric and capillary electrophoresis-based methods, as well as theoretical and computational estimates. In a spectrophotometric study of warfarin sodium with a reported pKa of 5.05 ± 0.1, it was noted that potentiometric determination is limited by the low solubility of the neutral form of warfarin [35]. Additionally, in the structure of warfarin, tautomeric forms can coexist based on the solvent composition, the open form (warfarin) and two diastereomeric hemiketals [5], which in varying proportions may contribute slightly to pKa shifts. Therefore, the pKa obtained via potentiometric titration represents a formulation-relevant estimate of the apparent pKa under specific solution conditions.

**Figure 3:**
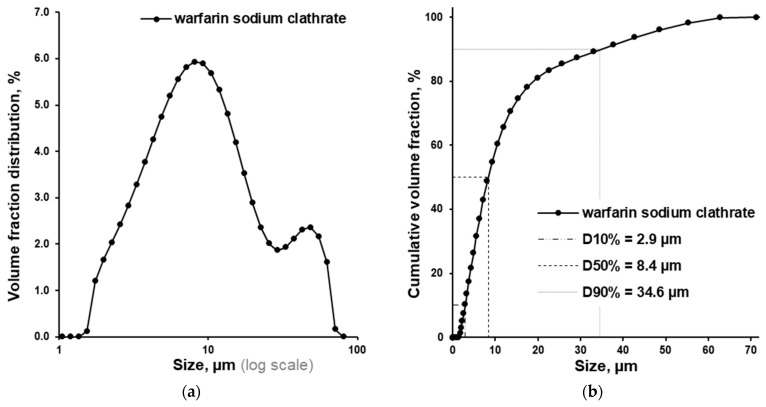
Volume fraction distribution (**a**) and cumulative volume fraction (**b**) of warfarin sodium clathrate (n = 3; R.S.D. < 5%).

Particle size distribution of warfarin sodium clathrate was determined in a previous study. D10%, D50%, and D90% comprised 2.9, 8.4, and 34.6 μm, respectively (Figure 3a,b) [4]. In this work, we obtained matrix pellets; thus, the particle size distribution additionally gives us indirect information about the pellet structure.

**Figure 4:**
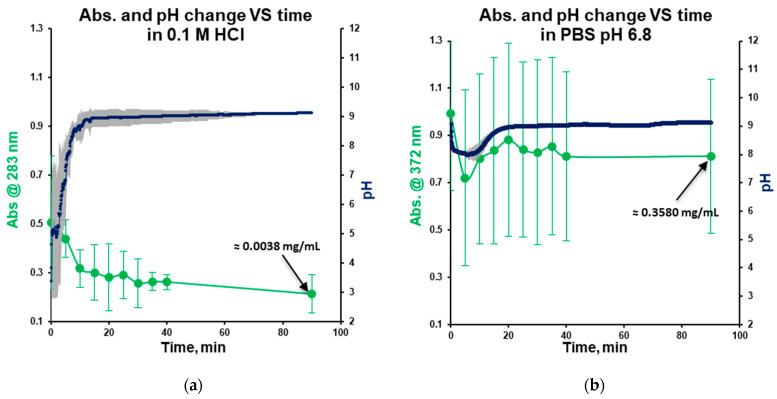

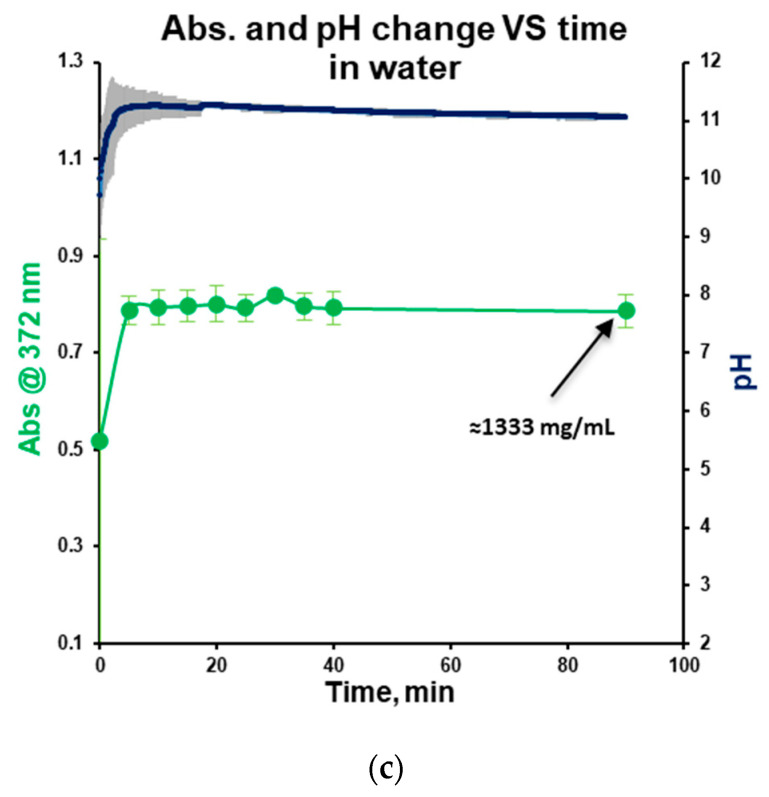
Observation of the UV absorbance and pH change during the dissolution of the raw drug substance in 0.1 M HCl (**a**), PBS with pH 6.8 (**b**), and water (**c**) (Av. ± S.D.; n = 3). In the 90 min point of measurements, the concentration of warfarin sodium was close to its equilibrium solubility in this medium (method in Section 2.2; UV spectrometer) and was mentioned on the graph.

Dissolution kinetics of warfarin sodium clathrate vary across the tested media. In stomach-simulated media (0.1 M HCl), concentration decreases as the pH increases (Figure 4a). This decrease in concentration is attributed to the low solubility of warfarin sodium in acidic conditions, where undissolved substances can act as crystallization centers, decreasing concentration. The concentration close to equilibrium was reached after 20 min. The equilibrium solubility of warfarin sodium in the form of clathrate in hydrochloric acid was determined at the level of 0.0038 ± 0.0006 mg/mL (Av. ± S.D.; method in Section 2.2).

In mouth-simulated media (PBS, pH 6.8), a slight decrease in pH and concentration was observed at the beginning of the dissolution, reaching saturation after 40 min (Figure 4b). The fluctuations in concentration and pH, indicated by substantial error bars, can be attributed to the time required for the buffer compensation of the pH change. The equilibrium solubility in PBS was established after 90 min at the level of 0.3580 ± 0.0578 mg/mL (Av. ± S.D.; method in Section 2.2).

In water, as the pH increases, both solubility and absorbance rise, reaching saturation at 20 min (Figure 4c), with an equilibrium solubility of 1335.325 ± 134.350 mg/mL (Av. ± S.D.; method in Section 2.2).

**Figure 5:**
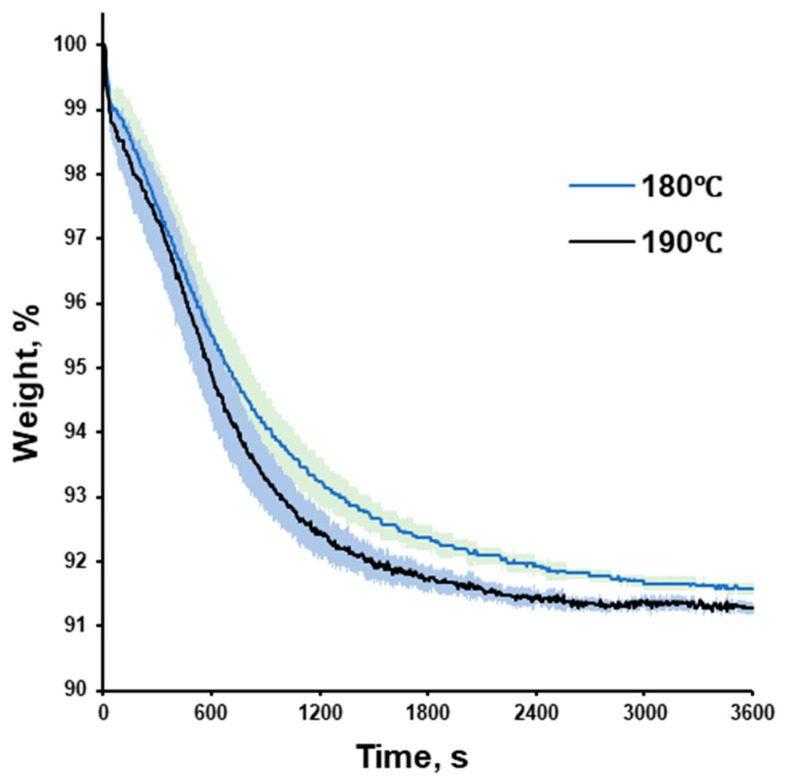
TGA profile of warfarin sodium clathrate exposed at 180 (n = 3) and 190 °C (n = 3) for 60 min (3600 s).

The thermal exposure of warfarin sodium clathrate at 180 and 190 °C for 60 min revealed a gradual weight loss (slower at 180 and faster at 190 °C), resulting in weight stabilization at the end of the experimental runs (Figure 5). The final weight loss during the TGA experiment corresponded to the solvated amount of isopropyl in warfarin sodium clathrate [6]. Thus, considering a relatively short residence time in the extrusion barrel and relatively fast cooling down of the filament right after extrusion, it can be expected that only a small portion of the isopropyl alcohol evaporates.

**Figure 6:**
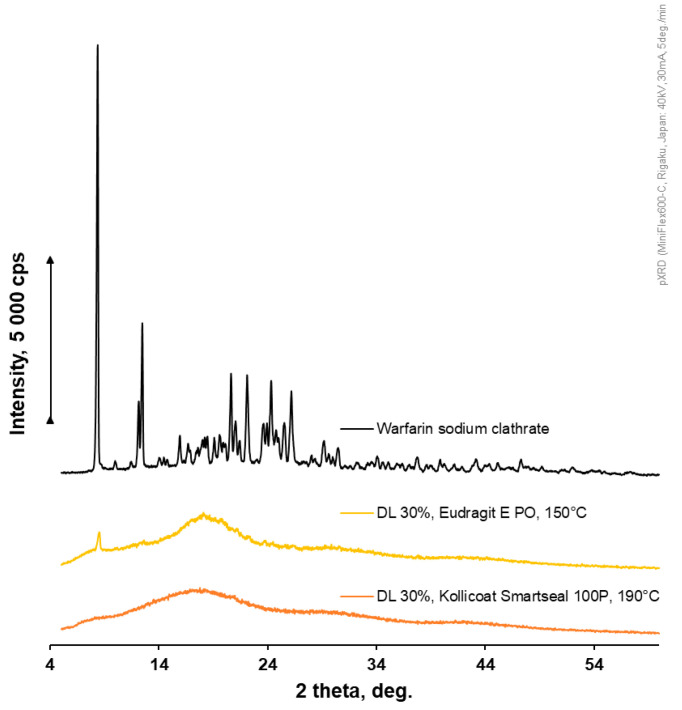
pXRD profiles of warfarin sodium clathrate and 30% drug-loaded (DL 30%) filaments based on Kollicoat^®^ Smartseal 100 P and Eudragit^®^ E PO after one year of storage at room temperature.

Drug-loaded filaments were characterized with pXRD. All extruded formulations, even at a higher drug loading of 30% and storage during one year at room temperature, were pXRD-amorphous (Figure 6). The extrusion conditions used allowed drug amorphization.

**Figure 7:**
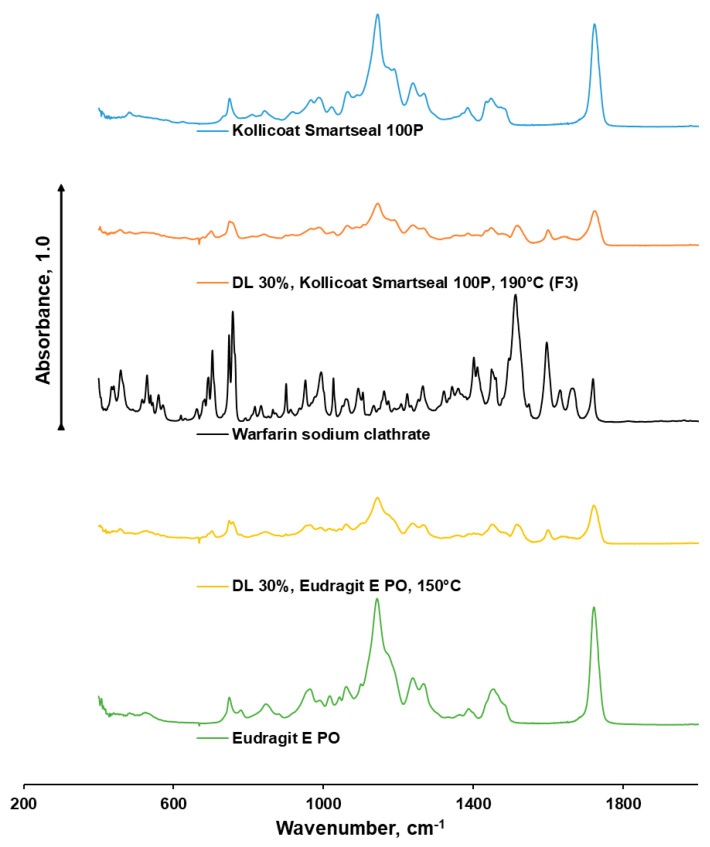
FTIR-ATR profiles of warfarin sodium clathrate and 30% drug-loaded (DL 30%) filaments based on Kollicoat^®^ Smartseal 100 P and Eudragit^®^ E PO after one year of storage at room temperature.

FTIR profiles of extruded formulations were different compared with the profile of the raw drug and polymers. No new peaks appeared, which suggested that no covalent interaction had happened (Figure 7).

The pure Kollicoat^®^ Smartseal 100 P showed characteristic ester carbonyl (C=O) stretching near 1730–1750 cm^−1^ and C–O–C vibrations around 1150–1250 cm^−1^. In the case of drug-loaded Kollicoat^®^ Smartseal 100 P, the warfarin peaks are significantly reduced or broadened, and the polymer’s carbonyl band shifts slightly. The shift in the C=O band can be explained by electrostatic attraction between the sodium ion and polymer oxygen atoms, while the reduction and broadening of warfarin’s characteristic peaks can be explained by hydrogen bonding between the carboxylate group of warfarin sodium and the ester or hydroxyl groups of Kollicoat^®^ Smartseal 100 P.

The pure Eudragit^®^ E PO also demonstrated characteristic ester carbonyl (C=O) stretching near 1730–1750 cm^−1^ and C–O–C vibrations around 1150–1250 cm^−1^ as well as similarity in terms of drug–polymer interaction. Apart from that, the FTIR profile revealed the evidence of tertiary amine N–CH_3_ groups in Eudragit^®^ E PO and tertiary amine band broadening in the warfarin-loaded Eudragit^®^ E PO formulation. This suggests an electrostatic attraction between the tertiary amine and warfarin’s acidic sites.

**Figure 8:**
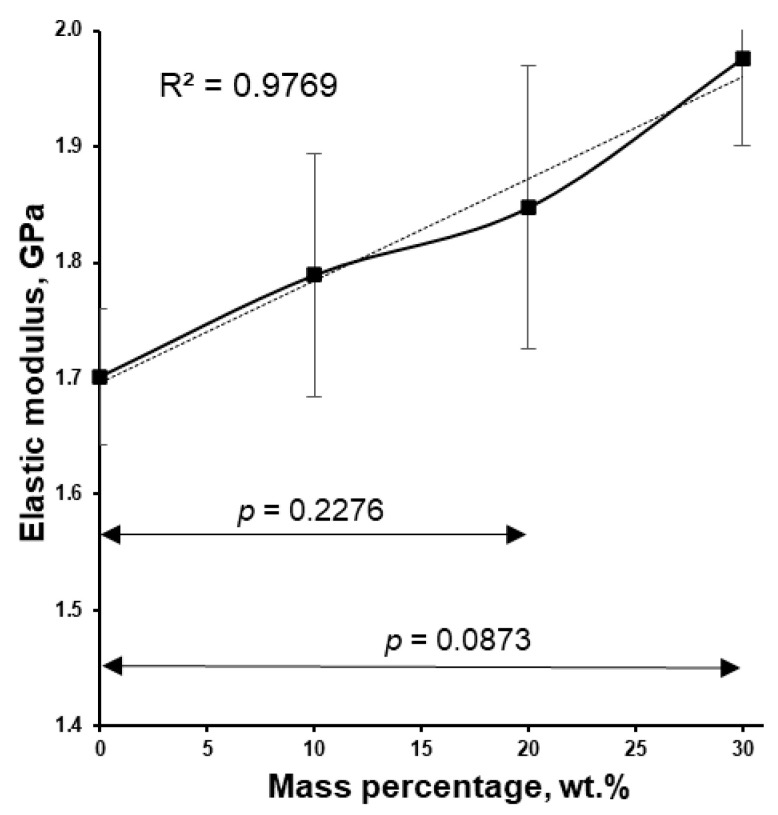
Filament elastic modulus dependence of the warfarin sodium clathrate in Kollicoat^®^ Smartseal 100 P drug loading (n = 5).

The filaments extruded were characterized by determining their elastic modulus (Figure 8). The elastic modulus of the samples linearly increased with the content of warfarin sodium clathrate inclusions. The stiffness of the warfarin sodium clathrate is probably higher than that of the polymer. As a result, an increase in the mass percentage of warfarin sodium clathrate leads to a higher elastic modulus (Figure 8). Typical mixture-rule behavior is observed for such a composition. The relationship between elastic modulus and drug loading for warfarin sodium clathrate filament samples is nearly linear (R^2^ = 0.9769).

**Figure 9:**
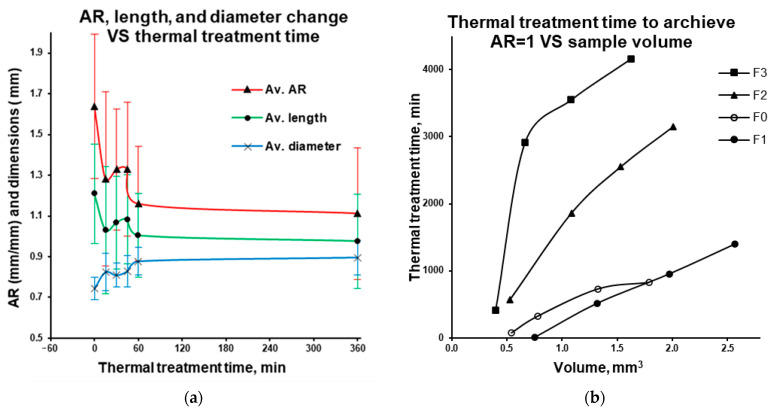
Averaged aspect ratio (AR), length, and diameter change as a function of the thermal treatment time (@ 80 °C bath) for the 20% drug-loaded Kollicoat^®^ Smartseal 100 P for 1 mm (n = 11) filament lengths (Av. ± S.D.; (**a**)). Thermal treatment time to achieve AR equal to 1 for formulations 1, 2, and 3 (F1, F2, and F3) and non-drug-loaded polymer filament (F0) in Kollicoat^®^ Smartseal 100 P as a function of the initial volume of the particle (**b**).

In all drug-loaded microparticles, thermal treatment of matrix pellets near the glass transition temperature results in a decrease in average particle length and an increase in average diameter, leading to an aspect ratio (AR) approaching 1. This trend is exemplified in Figure 9a for 20% Kollicoat^®^ Smartseal 100 P drug-loaded microparticles with an initial length of 1 mm. During thermal treatment near the polymer’s glass transition temperature, the polymer macromolecules in the microparticle undergo elastic deformation, reducing the “frozen” strain caused by filament pulling after the HME process. However, as drug loading increases, this deformation becomes restricted due to warfarin sodium clathrate inclusions. Drug substance influenced the structure and weight fraction of polymer in the matrix system, thus decreasing the “frozen” strain and relaxations upon postprocessing thermal treatment. Extrapolation of the average length and diameter of treated particles (at drug loadings of 10, 20, and 30 wt.% (Figure 9b) exemplified for Kollicoat^®^ Smartseal 100 P 20% drug loading for 1 mm particles in Figure 9a) allows for the determination of the required thermal treatment time to achieve an AR of 1.

Considering that, upon thermal treatment, the relaxation of the polymer is happening in a specific volume, the required time to achieve the AR of 1 was plotted versus the calculated volume of particles (Figure 9b). As shown, smaller initial microparticles and microparticles with higher polymer content (lower drug load) require shorter thermal treatment time to achieve a desirable AR. As the drug loading increases, the thermal treatment time required to achieve a particle with AR = 1 rises significantly. For particles ranging from 1 to 2 mm in length, 360 min of thermal treatment is sufficient to achieve an AR of 1. However, for larger particles, treatment beyond 360 min is necessary, which is unfavorable.

**Figure 10:**
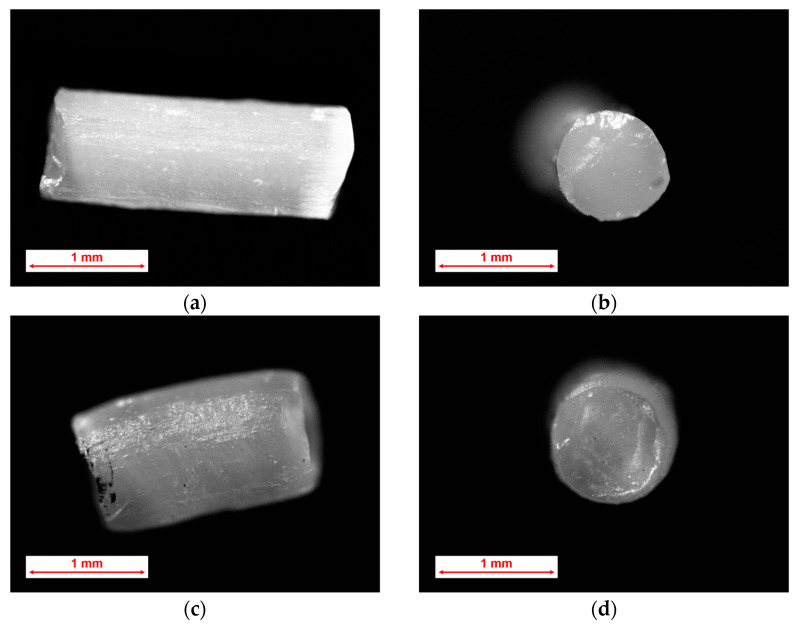
Example of particle microscopy (20 wt.% of warfarin sodium clathrate in Kollicoat^®^ Smartseal 100 P) before (**a**,**b**) and after (**c**,**d**) 360 min of thermal treatment. Magnification 4× (picture size: 6.4 × 8.53 cm).

Along with the changes in length and diameter during thermal treatment, the ‘smoothing’ of sharp edges also occurs. While this ‘smoothing’ is not visually apparent as compared with other polymers [24], it does take place, particularly at the sharp edges, which is exemplified by 20% drug-loaded microparticles for Kollicoat*^®^* Smartseal 100 P (Figure 10) and for Eudragit^®^ E PO. Another observable effect of thermal treatment is the increase in surface roughness, likely caused by polymer shrinking.

**Figure 11:**
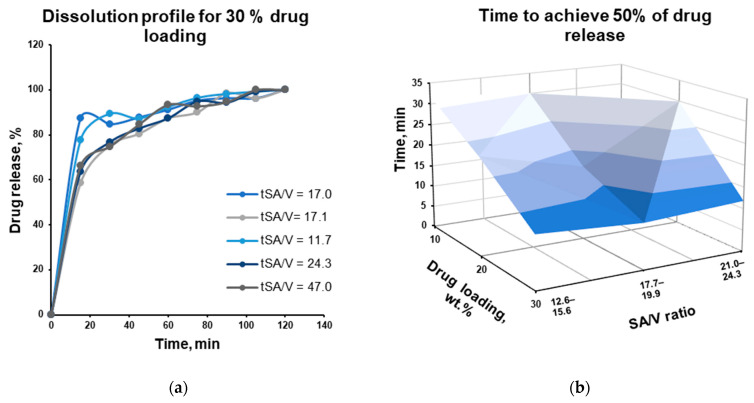
The dissolution profile of 30% drug-loaded Kollicoat^®^ Smartseal 100 P filament particles in 0.1 M HCl solution (**a**) and the time needed to achieve 50% of the drug release for Kollicoat^®^ Smartseal 100 P microparticles as a function of drug loading and surface-area-to-volume ratio (SA/V, cm^2^/cm^3^; (**b**)).

The drug release from thermally treated microparticles with the highest DL of 30% and with a close-to-one aspect ratio was investigated. No or insignificant drug release (non-quantifiable concentration) during at least the first 5 min of the dissolution test in oral cavity-simulating media (PBS pH 6.8) confirmed the taste-masking function of the Kollicoat^®^ Smartseal 100 P polymer in matrix microparticles. Samples with higher SA/V ratios are expected to exhibit the highest dissolution rates in 0.1 M HCl solution due to the higher surface area available for drug dissolution. However, the effect of the SA/V ratio was pronounced (exemplified by Figure 11a and shown in Figure 11b). This can be explained by the relatively narrowly investigated SA/V range of the obtained microparticles. The heterogeneity of the samples can also be considered as a contributing factor. Meanwhile, the increase in drug loading showed a clear increase in drug release, such as the time required to reach 50% drug release (Figure 11b).

If at least 80% of the drug is released in 30 min, it can be considered an immediate-release (IR) dosage form [36]. Drug release reaches at least 80% in most cases after 60 min for Kollicoat*^®^* Smartseal 100 P microparticles, indicating that this warfarin formulation is not a good fit for an IR dosage form, while the negligible dissolution in PBS confirms the concept of the taste-masking approach.

## 4. Conclusions

The preparation of taste-masked matrix pellets by HME and the ‘smoothing’ of their sharp edges by thermal postprocessing remain new and under investigation. This study first demonstrated the potential of Kollicoat^®^ Smartseal 100 P for this process, exemplified by warfarin sodium-containing microparticles. After thermal treatment of the microparticles, the edges become ‘smoother’, contributing to a more rounded particle shape, which should be beneficial for mouthfeel and medical compliance. At 0.8 mm of HME filament diameter, the preferred filament cutting lengths range from 1 to 2 mm, because longer initial lengths result in extended thermal treatment duration to achieve the desirable close-to-one aspect ratio. By varying the drug loading and matrix pellet size, different dosing options can be achieved. While the dependence on drug loading in the matrix pellets was clearly observed, the influence of the SA/V ratio was not pronounced in the tested SA/V ratio range. Particles prepared from the HME filament with smaller diameters can provide a higher SA/V ratio and faster dissolution, which leaves room for further investigation. This study showed the opportunity for the preparation of taste-masked matrix pellets based on a specific taste-masking polymer in order to obtain the microparticles without sharp edges and improved mouthfeel and compliance.

## Data Availability

The data presented in this study is available upon request from the corresponding author.
